# Endoscopic Image-Based Skill Assessment in Robot-Assisted Minimally Invasive Surgery

**DOI:** 10.3390/s21165412

**Published:** 2021-08-10

**Authors:** Gábor Lajkó, Renáta Nagyné Elek, Tamás Haidegger

**Affiliations:** 1 Autonomous Systems Track, Double Degree Programme, EIT Digital Master School, Technische Universität Berlin, Straße des 17. Juni 135, 10623 Berlin, Germany; gabor.lajko@masterschool.eitdigital.eu; 2 ELTE Faculty of Informatics, Pázmány Péter Sétány 1/C, Eötvös Loránd University, Egyetem tér 1-3, 1117 Budapest, Hungary; 3 Antal Bejczy Center for Intelligent Robotics, University Research and Innovation Center, Óbuda University, 1034 Budapest, Hungary; haidegger@irob.uni-obuda.hu; 4 Doctoral School of Applied Informatics and Applied Mathematics, Óbuda University, Bécsi út 96/b, 1034 Budapest, Hungary; 5 John von Neumann Faculty of Informatics, Óbuda University, Bécsi út 96/b, 1034 Budapest, Hungary; 6 Austrian Center for Medical Innovation and Technology, Viktor Kaplan-Straße 2/1, 2700 Wiener Neustadt, Austria

**Keywords:** robot-assisted minimally invasive surgery, surgical skill assessment, JIGSAWS, optical flow

## Abstract

Objective skill assessment-based personal performance feedback is a vital part of surgical training. Either kinematic—acquired through surgical robotic systems, mounted sensors on tooltips or wearable sensors—or visual input data can be employed to perform objective algorithm-driven skill assessment. Kinematic data have been successfully linked with the expertise of surgeons performing Robot-Assisted Minimally Invasive Surgery (RAMIS) procedures, but for traditional, manual Minimally Invasive Surgery (MIS), they are not readily available as a method. 3D visual features-based evaluation methods tend to outperform 2D methods, but their utility is limited and not suited to MIS training, therefore our proposed solution relies on 2D features. The application of additional sensors potentially enhances the performance of either approach. This paper introduces a general 2D image-based solution that enables the creation and application of surgical skill assessment in any training environment. The 2D features were processed using the feature extraction techniques of a previously published benchmark to assess the attainable accuracy. We relied on the JHU–ISI Gesture and Skill Assessment Working Set dataset—co-developed by the Johns Hopkins University and Intuitive Surgical Inc. Using this well-established set gives us the opportunity to comparatively evaluate different feature extraction techniques. The algorithm reached up to 95.74% accuracy in individual trials. The highest mean accuracy—averaged over five cross-validation trials—for the surgical subtask of Knot-Tying was 83.54%, for Needle-Passing 84.23% and for Suturing 81.58%. The proposed method measured well against the state of the art in 2D visual-based skill assessment, with more than 80% accuracy for all three surgical subtasks available in JIGSAWS (Knot-Tying, Suturing and Needle-Passing). By introducing new visual features—such as image-based orientation and image-based collision detection—or, from the evaluation side, utilising other Support Vector Machine kernel methods, tuning the hyperparameters or using other classification methods (e.g., the boosted trees algorithm) instead, classification accuracy can be further improved. We showed the potential use of optical flow as an input for RAMIS skill assessment, highlighting the maximum accuracy achievable with these data by evaluating it with an established skill assessment benchmark, by evaluating its methods independently. The highest performing method, the Residual Neural Network, reached means of 81.89%, 84.23% and 83.54% accuracy for the skills of Suturing, Needle-Passing and Knot-Tying, respectively.

## 1. Introduction

### 1.1. Background

Minimally Invasive Surgery (MIS) is a collection of surgical techniques that aim to limit the size of incisions and tissue trauma, in order to decrease the recovery time, the inflicted pain and the risk of infections during surgeries. The introduction of MIS has revolutionised operations in the past 50 years [1]. Twenty-five years ago, with the introduction of robotic telesurgical systems, a new form of MIS was born: the Robot-Assisted Minimally Invasive Surgery (RAMIS) [2,3].

External sensors such as sensory gloves have been used to evaluate surgical skill in open surgery [4]. Similarly, for MIS, mounted flex sensors have been employed [5] or the Fundamentals of Robotic Surgery (FRS) Dome was used to gather data [6].

However, with smaller incisions, navigating the surgical tools becomes more challenging. The increasing quality of endoscopic imaging systems has been an enabler of MIS. These cameras offer real-time visual feedback, effectively replacing the lost direct visual information surgeons used to have in traditional invasive surgeries. MIS requires extensive skills, so that surgeons are able to master the specifically designed precision tools. Multiple publications [7,8,9,10] indicate that the use of these tools in tandem with the endoscopic camera results in sub-optimal ergonomics, due to the limitations of human joints or because of the increased complexity in hand-eye coordination. The monitoring screen is also hard to place ergonomically. In MIS—where the 2D camera is handled by an assistant—the surgeon has to take a leading position, coordinating the work of a surgical team, while having to be focused and precise, and keeping an eye on the 2D feedback screen [11].

The main advantages of RAMIS are two-fold: it brings benefits to both the patient and the surgeon. On the patient’s side, the higher precision of the surgery results in less damage to surrounding healthy tissues and nerves, causing significantly less pain and shorter recovery time [12]. For the surgeons, RAMIS provides a significantly better visual overview of the operating area thanks to the high definition 3D camera systems employed. The robotic arms also offer superior dexterity, with a larger range of motion than that of the human hand, being able to rotate a full 360 degrees, enabling the surgeon to perform tasks in ways impossible with manual tools. This also means that, with the robotic arm, the surgeon has easier and safer access to hard-to-reach areas during a surgery while also receiving a more detailed, 3D visual feedback in an ergonomically superior environment to that of the traditional MIS. All this combined enables the surgeon to keep a better focus, be more comfortable during the operation and, with less stress, perform better [13].

RAMIS is not without disadvantages, however. In traditional MIS, primarily human errors occur, and, although the equipment may malfunction during MIS, the chances of malfunction are significantly higher for RAMIS. Surgical robot systems—such as the market-leading da Vinci Surgical System [14]—are complex structures built of many parts—actuated sensors, multiple specialised robotic joint configurations, precise surgical end-effector tools, etc.—all of which are subject to mechanical failure; therefore, it is paramount that the robotic equipment be maintained and subjected to near-constant mechanical supervision. The electrical system is a critical component as well, which may cause burns in the case of failure. Robotic surgeries may also take longer, provided there is not enough capacity, or there is a lack of sufficiently experienced robot surgeons. RAMIS systems used in clinical practice need to meet high safety standards. Out of more than 1.7 million robotic procedures in the US alone, in less than 0.6% of the cases do adverse events occur [15], 75.9% of which are due to mechanical failure, on average constituting between 0.9% and 3% of procedures overall [15]. The most common practice in the case of any such issue with RAMIS procedures is to convert the procedure to non-robotic techniques, such as traditional MIS or even open surgery. Other options include rescheduling the operation and restarting the system in order to be able to continue.

MIS requires multiple years of training, but, even though its benefits are beyond doubt, frequent skill assessment of the trainees is still not a part of the clinical practice [16]. Standard evaluation techniques include checking the time trainees take to perform tasks, having an expert surgeon overseeing the exams or providing expert rating based on pre-defined criteria, such as GEARS [17] or OSATS [18], both of which are validated metrics, proven to correlate with surgical skills. Since traditional surgical skill assessment techniques often require the expertise of skilled surgeons, whose time is a valuable and scarce resource, the need for automatic skill assessment techniques is given [19,20].

Automated skill assessment aims to free up expert evaluators by reliably automating the process of assessment as much as possible. It can classify the expertise level of surgeons-in-training or provide personalised feedback and precise insight on how to improve certain subtasks, with the use of sensors specifically set up for each subtask. In principle, they indirectly support patient care by supporting the training of surgeons, without overburdening the trainees with additional equipment. The problem at hand is that no reliable automatic skill assessment technique has been proven to be applicable to both MIS and RAMIS.

### 1.2. Literature Review

Either kinematic or visual data are used primarily [21]. Kinematic solutions are more commonly used [21], but they cannot be used for the training of traditional, manual MIS. Given the popularity of deep learning methods in this field of research, the issue of overfitting and thereby the lack of generalisation capability is also an ever-present issue, often combated by cross-validation, regularisation or transfer learning [22].

The use of kinematic data predates visual data in the field of surgical skill assessment and offers very precise and useful assessment possibilities. In general, kinematic data are the most suitable for surgical skill assessment in RAMIS procedures, as the data come directly from the internal sensors of each joint. Fawaz et al. achieved 100% accuracy with both classification and regression tasks performed on the kinematics data of JIGSAWS, using a combination of Approximate Entropy (ApEn) and Fully Connected Convolutional Neural Networks (FCN) [23], providing tangible, personalised feedback for surgeons in training. However, kinematics is not indubitably and ultimately superior to visuals anymore [24]. Funke et al. achieved 100% accuracy in classification using 3D visual features [25]. However, during manual MIS training, where assessment methods would be the most useful, there is no direct access to the kinematic data, unless directly mounted tooltip and hand motion sensors are applied, the use of which may be invasive to trainees and might interfere with the freedom of their movement. Similarly, the use of 3D features is often hindered due to the lack of proper stereo camera equipment. Therefore, our focus was investigating the utility of 2D features, specifically 2D sparse optical flow.

Ming et al. achieved a mean accuracy of 79.29%/76.79%, 80.71%/83.81% and 72.57% /76.65% on the basis of Space Temporal Interest Points (STIP)/Improved Dense Trajectory (iDT) representation of the three subtasks of JIGSAWS, respectively [26]. The STIP method is built on similar theoretical bases as our sparse optical flow-based solution, given that both of them track the movement of points over time. Therefore, their results are ideal for comparative analysis of our own. They found that, although iDT produced better results, its use is way more memory demanding, and therefore not practical. They proved that, similar to kinematic-based solutions, it is possible to distinguish novice and expert users based on visual features. Their solution relies on a histogram of features, processed by a Support Vector Machine (SVM), to achieve classification.

Frequency-based solutions, such as Discrete Fourier Transform (DFT) and Discrete Cosine Transform (DCT), are commonly employed [27], and, although they are usually outperformed by Deep Neural Networks (DNN), Zia et al.’s work is a notable exception [28]. Sequential motion texture (SMT) and ApEn are also utilised [28]. Out of deep learning methods, Convolutional Neural Networks (CNN), Recurrent Neural Networks (RNN) and FCNs are the most frequently used, with other architectures such as Long Short-Term Memory (LSTM), VGG and Residual Neural Networks (ResNet) also being used for classification [21]. Out of CNN, RNN and FCN, CNN seems to be the superior technique, due to the lack of long sequences in surgical data, which recurrent networks need, and because the generally employed statistical features eliminate the use of temporal data that methods such as LSTM would require [21]. The most commonly used input sources are hand and tool motion sensors and videos [21].

### 1.3. Objective

Visual-based methods are more practical in surgeon training, as kinematic data are only available in RAMIS (or with the use of external sensor system), while visual data are accessible in MIS as well, and they require no explicit preparation once they are trained and cross-validated [21]. Our goal was to develop an objective, automated and validated surgical skill assessment methodology which can be applicable both MIS and RAMIS. Since kinematic data—which are an input in most of the developed automated skill assessment methods—without additional sensors are only available RAMIS, for general (MIS and RAMIS) studies, RAMIS-recorded datasets can be a reliable ground truth. Furthermore, in this study, only the endoscopic image data were utilised for the skill assessment. Employing 3D visual features, which is only available in RAMIS, has been shown to perform similarly well to kinematics-based skill assessment methods. However, in order to help the integration of automated skill assessment into surgical training practices not just in RAMIS but in MIS as well, we opted for 2D visual features, aiming to demonstrate their efficacy in comparison to similar state of the art solutions.

### 1.4. Contributions

**Figure 1:**
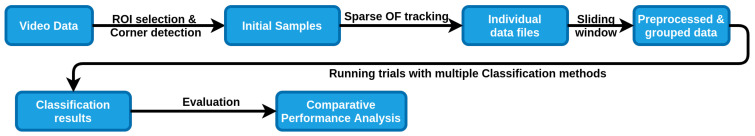
The proposed workflow: First, we compute and save the initial samples from each video input using user-selected regions of interest. Then, by tracking them with the sparse Lucas–Kanade OF—tracking the movement of both surgical tools independently—we process the videos, creating data files that are further processed by a sliding window method, outputting the final input data. Using several different classification methods, we determine the users’ expertise.

This article introduces a widely applicable, scalable and practical assessment data generation method, aiming to enable the integration of visual-based surgical skill assessment solutions into the curriculum of MIS trainings, by providing generally accessible 2D visual features. Besides introducing the results of multiple feature extraction techniques used for classification, we include a detailed performance analysis in the Appendix B. Figure 1 illustrates the overall workflow of our proposed solution. Additional related resources such as the source codes, result and data files are publicly available on Github (https://github.com/ABC-iRobotics/VisDataSurgicalSkill, access date on 30 June 2021.).

## 2. Materials and Methods

### 2.1. Dataset

**Figure 2:**
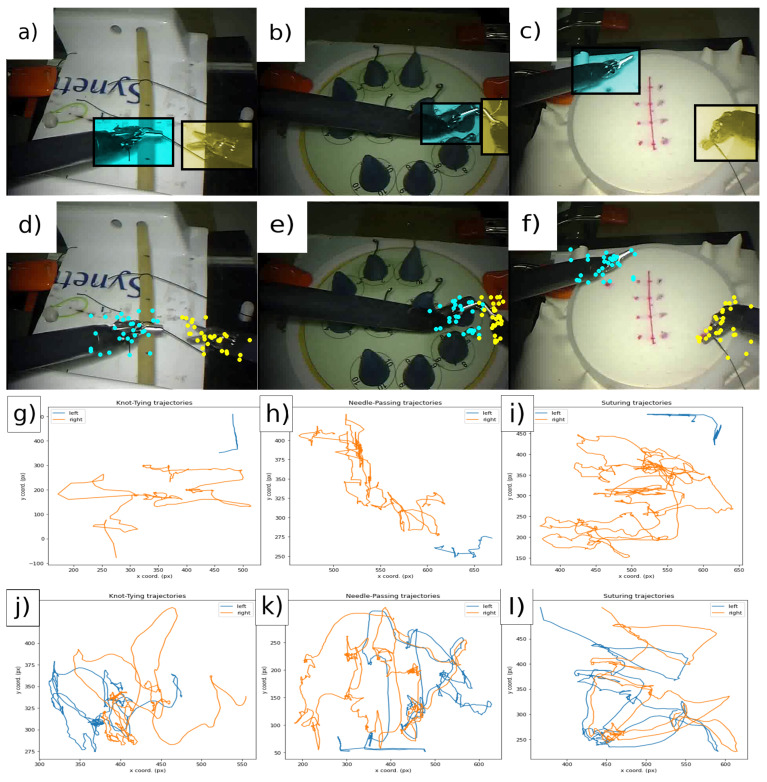
The three surgical tasks in the JIGSAWS database: Knot-Tying (KT), Needle-Passing (NP) and Suturing (ST). (**a**–**c**) The selection of Regions Of Interest (ROIs). (**d**–**f**) The initial samples computed by the Shi–Tomasi method. The third and fourth rows show one tracked points’ trajectory from each tool (blue, left; orange, right). (**g**–**i**) The data are from a novice user. (**j**–**l**) The data are from an expert subject.

Created by the cooperation of the Johns Hopkins University (JHU) and the creators of the da Vinci Surgical System [14], Intuitive Surgical, Inc. (ISI), the JHU–ISI Gesture and Skill Assessment Working Set (JIGSAWS) [29] is one of the most widely used surgical skill assessment datasets—as evidenced by Yanik et al. [21]. It is also the only publicly available annotated dataset for surgical skill assessment. It contains kinematic and video data on three basic surgical tasks (Knot-Tying, Suturing and Needle-Passing)—essential in surgical training curricula—and comes annotated with expert rating scores by an experienced surgeon [30]. For our purposes, each video is labelled according to the expertise level of the performing surgeon for supervised learning, but it is also possible to use the GEARS score [17] of the annotation to provide more complex, regression-based evaluations. Figure 2 illustrates the three main steps of our workflow (selecting regions of interest, saving initial samples and tracking trajectories) on all three surgical tasks (Knot-Tying, Suturing and Needle-Passing).

### 2.2. Optical Flow

Optical Flow (OF) is a fundamental algorithm for movement detection in video analysis. It estimates motion between two consecutive video frames by calculating a shift vector to quantify the displacement difference [31]. OF generally corresponds to the motion field, with some exceptions. The Lucas–Kanade method is commonly used to calculate the OF for a sparse feature set. The main idea of this method is based on a local motion constancy assumption, where nearby pixels have the same displacement direction. OF is an established algorithm in surgical skill assessment [25]. Our proposed method uses the Pyramidal Implementation of the Lucas–Kanade algorithm [32].

Since optical flow is a well-known motion estimation technique in computer vision, in-depth mathematical presentation of the topic and the different approaches is out of the scope of this paper. The fundamental assumption of optical flow is that the pixel intensities do not change between two frames (brightness constancy):

(1) $$\begin{array}{c} I(x,y,t)=I(x+dx,y+dy,t+dt) \end{array}$$

where *x* and *y* are the pixel coordinates, *dx* and *dy* are the pixel displacement, *t* is the time and dt is the elapsed time. From the Taylor expansion of this basic equation, the optical flow constraint equation can be derived:

(2) $$\begin{array}{c} I_{x}v_{x}+I_{y}v_{y}+I_{t}=0 \end{array}$$

where $I_{x}$, $I_{y}$ and $I_{t}$ are the derivatives of the image function I(x,y) with respect to *x*, *y* and *t*; vector $V=(v_{x},v_{y})$ defines the velocity vector in *x* and *y* direction. This equation is called the optical flow equation, which is under-determined. To solve the equation, other assumptions are necessary. In the case of Kanade–Lucas OF, this assumption is that the a small region of the pixels have the same velocity [33].

### 2.3. Classification Methods

We used the codebase of Anh et al. [34]—available on Github (https://github.com/SimonNgj/compssa, access date on 30 June 2021.)—to perform classification. Taking the kinematic data of the JIGSAWS set as input [29], they implemented 9 different evaluation methods to provide a benchmark for skill assessment solutions, categorising surgical skills into expert, intermediate and novice classes. Our solution uses some of the same methods from this codebase, repurposed for 2D visual input features. Section 2.3.1, Section 2.3.2, Section 2.3.3, Section 2.3.4, Section 2.3.5 and Section 2.3.6 introduce these methods.

To counteract overfitting—i.e., a higher model complexity than that of the data themselves, learning outliers and noise as part of the pattern, thereby losing on generalisation capability—the LOSO (Leave-One-Super-Trial-Out) cross-validation technique was used, along with L2 regularisation for each of the benchmark methods introduced below. Same padding is used in most places, to keep the dimensions of the output.

(3) $$\begin{array}{c} output\_width=\frac{W-F_{w}+2P}{S_{w}}+1 \end{array}$$

(4) $$\begin{array}{c} output\_height=\frac{H-F_{h}+2P}{S_{h}}+1 \end{array}$$

where *W* and *H* are the width and height of input, *F* are filter dimensions and *P* is the padding size (i.e., the number of rows or columns to be padded). In the case of same padding, the following stands:

(5) $$\begin{array}{c} output\_height=ceil(\frac{H}{S_{h}}) \end{array}$$

(6) $$\begin{array}{c} output\_width=ceil(\frac{W}{S_{w}}). \end{array}$$

#### 2.3.1. Convolutional Neural Network

Commonly used for classification, segmentation and image processing, Convolutional Neural Networks (CNNs) ensure translation invariance and parameter sharing through convolution (essentially specialised sliding filter operations) [35,36]. They are based on the assumption that nearby data are more closely correlated than further ones. In our data (see Section 2.5), the two surgical tools are separated, therefore closer data are more likely to belong to the same tool. CNN relies on such local dependencies, and in our case this is further supported by the the arrangement in which the optical flow displacement values—describing speed in relation to the frame rate—and the Position pixel-coordinates are side-by-side, with the rows corresponding to timestamps/frames.

The network uses two one-dimensional convolutional layers with the Rectified Linear Unit (ReLU) activation functions, introducing non-linearity to the system with the equation ReLu(z)=max(0,z) followed by batch normalisation layers. Then, a one-dimensional Global Average Pooling (GAP) is applied to reduce data complexity and avoid overfitting by reducing the total number of parameters in the models. Ultimately, a dense layer with softmax activation ($softmax\left(z_{i}\right)=\frac{exp(z_{i})}{\Sigma_{j}exp\left(z_{j}\right)}$) is used to create the output as a vector of three, corresponding to the three classes of classification.

#### 2.3.2. Long Short-Term Memory

Long Short-Term Memory networks (LSTMs) are specialised neural networks by design, ideal for time series data analysis [37]. Using the combination of three types of gates (input, output and forget) and a dedicated memory cell—storing the internal representation of the learned information—they are able to record long term data representations. Their use is beneficial in surgical skill assessment, where the sequentiality of actions and precision movements is relevant for the successful execution of surgical subtasks. Suitable subtask-segmentation may be required, in order to avoid the confusion of patterns belonging to different sequences.

The input gate takes the input from the current time-stamp, $x_{t}\in R^{N}$, the output from the previous LSTM-unit, $h_{t-1}$, and the previous memory cell, $c_{t-1}$. LSTMs make use of the sigmoid activation function ( $\sigma \left(z\right)=\frac{1}{1+e^{-z}}$, bounded between [0,1] ) to process the input information:

(7) $$i_{t}=\sigma \left(W_{xi}x_{t}+W_{hi}h_{t-1}+W_{ci}c_{t-1}+b_{i}\right).$$

Forget gates serve as a sort of selection method. This one layer neural network determines whether the given data should be kept or removed from the current internal state:

(8) $$f_{t}=\sigma (W_{xf}x_{t}+W_{hf}h_{t-1}+W_{cf}c_{t-1}+b_{f}.$$

The learned knowledge is stored in memory cells. These cells are updated by combining (⊙) the memory cell with the new information:

(9) $$c_{t}=f_{t}⊙c_{t-1}+i_{t}⊙tanh\left(W_{xc}x_{t}+W_{hc}h_{t-1}+b_{c}\right).$$

Finally, the output gate controls the information passed onto the next LSTM block:

(10) $$h_{t}=o_{t}⊙tanh\left(c_{t}\right),\;o_{t}=\sigma \left(W_{xo}x_{t}+W_{ho}h_{t-1}+W_{co}c_{t}+b_{o}\right).$$

#### 2.3.3. CNN + LSTM

A straightforward combination of the previous two models, namely two convolutional layers—batch optimisation and ReLu activation functions—followed by two LSTM blocks, CNN + LSTM is a slightly more complex neural network architecture. Among others, Li et al. [38] demonstrated high accuracy predictions using the combination of these methods. The temporal information of the convolutional layers’ outputs is processed by the LSTM blocks, in order to learn contextually, but from an already processed information source.

#### 2.3.4. Residual Neural Network

Primarily used for classification tasks, using so-called skip connections, as shortcuts to solve the degradation problem [39]—essentially short circuiting shallow layers to deep layers—Residual Neural Networks (ResNets) enable the creation of deeper networks without loss of performance. It is a reliable technique even within smaller networks. The model of the benchmark also consists of only 3 blocks, meaning that it does not utilise its strength to the fullest (given that there are not many layers to skip), but it still can perform accurate classification.

#### 2.3.5. Convolutional Autoencoder

Traditionally used for data compression, dimensionality reduction and the denoising of data without significant information loss, an autoencoder—often symmetric, built up of two blocks, an encoder and a decoder—is an unsupervised machine learning model [40]. The encoder aims to create a copy of its input as an output, reverse engineering the problem by trying to find the right filter. The autoencoder created by Ahn et al. [34] is built using convolutional layers. It is also common to use fully connected layers. The encoder compresses the input time series into a latent space representation, and then the network tries restructuring it into the original input data in the decoder. For classification, after the network has been fully trained, the encoder’s output is fed to an SVM classifier.

#### 2.3.6. Frequency Domain Transformations

The Discrete Fourier Transformation and the Discrete Cosine Transformation are traditionally used to transform time series data from the time domain to the frequency domain. The use of frequency features in surgical skill assessment was proven to perform well by Zia et al. [27].

The main assumption of these techniques is that the more competent a user is in the given skill, the smoother and more predictable the time series representation will be. The initial $X\in R^{N\times L}$ time series, where *N* is the feature size (240) and *L* is the sample size (the given videos’ frame number), is initially separated into univariate time series, the frequency coefficients of which are calculated and concatenated with a frequency matrix $Y\in R^{N\times L}$.

After computing matrix Y, iterating through each row, the highest peaks are kept, while the others are discarded. With F<L being the number of peaks kept, we get the reduced matrix $\hat{Y}\in R^{N\times F}$. The F highest peaks represent the most significant frequencies of the input segment. Classification is achieved by converting $\hat{Y}$ into a one-dimensional matrix.

The two methods only differ essentially in the frequency matrix used:

(11) $$\mathrm{DFT}:\;Y_{k}=\Sigma_{t=0}^{L-1}x_{l}\times e^{-\frac{i2\pi kl}{L}},$$

(12) $$\mathrm{DCT}:\;Y_{k}=\frac{1}{2}x_{0}+\Sigma_{l=1}^{L-1}x_{l}\times cos\left[\frac{\pi }{L}l\left(k+\frac{1}{2}\right)\right].$$

The periodicity of DFT breaks the continuity, while DCT is fully continuous.

### 2.4. Setup

The computation of results was performed in a 64 bit Ubuntu 18.04.5 LTS environment, using jupyter-notebooks with Python 3.6.9, and the following packages: opencv-python 4.2.0.34, scikit-learn 0.23.2 and Keras 2.1.5. The computer’s CPU is a twelve-core Intel^®^ Core™ i7-8750H CPU, running on 2.20 GHz with 8 GB RAM and a GeForce GTX 1050 Ti/PCIe/SSE2 GPU.

### 2.5. Data Generation

The Kanade–Lucas OF requires the output of the Shi–Tomasi Corner Detection [41], published in 1994—which is built on the Harris corner detection [42] from 1988, with the additional criteria of filtering for “good features to track”, and, since then, it has become one of the most widely used corner detection methods over the world.

As a first step, a suitable initial frame is found, where both surgical tools are visible, and then user-selected regions of interest are preprocessed and saved. The frames are first turned gray-scale, then blurred by a median filter, and ran through a binary filter using adaptive thresholds, in order to denoise the frames and enable the better detection of features, using the Shi–Tomasi detector on the respective ROIs.

The resulting output constitutes the initial features to be tracked. Each video is traversed, set to the initial frame, the features of which are then tracked by the Kanade–Lucas OF. The features are extracted from each frame of the video and collected in a list, with the dimensions: frame_number×sample_size×2×2. This list is then iterated through, and for each frame’s data a row of 240 features—made up of the respective OF and positional information of each tool—is added to the output. No other parameters or metrics (acceleration, jerk, etc) were computed. As our solution performs an automated global assessment, we do not need to rely on the separation of gestures within the subtasks. We evaluate the expertise level based on the whole procedure performed by the subject.

Given the ROI data and the generated output, grouping the data according to the surgical tasks and the expertise level of users constitutes the final output. This is accomplished using the sliding window preprocessing method implemented by Anh et al. for their benchmark [34], to process the multivariate time series and separate chunks of the data into uniformly sized local windows, thereby enabling the evaluation of our data with the same networks originally designed for the kinematic data of JIGSAWS.

## 3. Results

We used the video data of the JIGSAWS dataset and evaluated the previously described benchmark methods. These data consist of recordings of eight subjects, with varying surgical experience, performing each of the three surgical subtasks (Knot-Tying, Needle-Passing and suturing) on the da Vinci Surgical System. Cross-validation is a strong tool against overfitting. To evaluate methods more precisely, it is customary to take the average of multiple cross-validated executions. As chosen by Anh et al. [34], each method was run five times for each generated input file. Within each run there were five trials, using the LOSO cross-validation method, and then the mean accuracy was calculated.

### 3.1. Intermediate Subjects

The intermediate skill class is prone to misclassification [21]. Funke et al.’s hybrid 3D network misclassified every intermediate surgeon into either expert or novice, using OF data for Knot-Tying [25]. This may partially be due to data disparity, as intermediate and expert subjects are underrepresented in comparison to novices [29]. Funke’s solution relied on a Leave-One-User-Out (LOUO) cross-validation, which is less robust to such disparity. Anh et al.’s benchmark also faced issues with the classification of intermediate users [34]. Table 1 shows the corresponding analysis of three-class classification confusion matrices. Given this, and seeing that other publications only distinguish between novices an experts as well, we decided to omit intermediate subjects from the main evaluation, and only comparatively analysed three-class classification, the result of which is presented in Table 2.

LSTM struggled with the classification accuracy even in the two-class version. With 78% of times not being able identify a single intermediate user, it is the worst performing method.

The Convolutional Autoencoder performed the best, with the rest of the methods forming a relatively balanced middle range. Every analysed method (other than the LSTM) could classify intermediate users with at least 66% accuracy.

### 3.2. Evaluation Metrics

The performance of each method is summed up in individual tables (found in Appendix A), with the following columns:

1. **Parameters:** The sliding window parameters used in the generation of the data being used for the given trials (window-size x step-size).
2. **Evaluation:** Model or SVM, the mode of accuracy calculation. Model means that the full trained model is used for the predictions, while SVM means that the last layer is removed and replaced with a Support Vector Machine classifier.
3. **Mean:** The average of the mean accuracies of given run. Five runs with three different skills means that there are 15 mean accuracies (calculated from the five cross-validation trials) and the average of these mean accuracies was taken for the predictions of both the model and the SVM.
4. **Standard Deviation:** On the same range of accuracies as above.
5. **Best run:** The highest mean accuracy—calculated from the five trials in the given run.
6. **Best trial:** The overall highest accuracy of every trial in that configuration.

In the case of the confusion matrices, the following expressions are used:

1. **Novice True Positive:** The user was a novice and classified as such.
2. **Novice False Positive:** The user was an expert but classified as a novice.
3. **Expert True Positive:** The user was an expert and classified as such.
4. **Expert False Positive:** The user was a novice but classified as an expert.

### 3.3. Results by Methods

#### 3.3.1. CNN

With a minimum standard deviation of 1.43% and best mean accuracy of 80.72%, CNN responded well to our data. Given that there are more novice users than experts, it is possible to achieve relatively high prediction accuracy by misclassifying experts, if the majority of novices are recognised. Therefore, it is important to analyse the confusion matrices of our methods.

Regarding the classification precision of CNN in particular, the corresponding confusion matrices showed that only in 0.92% of the cases (11 out of 1200 matrices) it did not find a single true positive for the novice class, and 15% (180 out of 1200) of the matrices had zero true positives for experts.

It can be concluded that the error is marginal, and the accuracy values seen in the summary table (Table A1) are reliable. It is also important to note that, although the SVM classification decreases the overall accuracy, it performs more consistently well overall. All 11 cases of zero true positive novice classification occurred in the model-based evaluation, as well as 168 out of the 180 for the experts. On the other hand, 183 out of 600 times during the model evaluation, there were zero false positives for experts, and 114 out of 600 times zero false positives for novices. The same numbers for the SVM results were 70 and 20. This is likely due to the fact that SVMs are susceptible to outliers. They perform well in high-dimensional feature spaces, and, although we have 240 features, the number of rows greatly outnumbers this (with row numbers between 1500 and 4000). Tuning hyperparameters—for example, C, to regulate the penalty for misclassified data—could increase the efficacy of the SVM classifier.

#### 3.3.2. LSTM

LSTMs are designed to learn patterns and model dependencies over time. Our data can be interpreted as a time-series, where each row (of 240 values) corresponds to one timestamp, so theoretically LSTM should be able to pick up on some patterns.

Out of 1200 confusion matrices (600 for model and SVM-based results each), 35 had zero true positives for novices (8 model-based, 27 SVM-based) and 756 had zero true positives for experts (400 model-based, 356 SVM based). The number of novice false positives was 49 (14 model-based, 35 SVM-based) and the number of expert false positives was 881 (493 model-based, 388 SVM-based). This is by far the worst performance out of all the compared methods. Table A2 shows the performance analysis of LSTM.

#### 3.3.3. CNN + LSTM

The CNN performed reliably, with a low standard deviation and a reasonable mean accuracy. LSTM on the other hand had up to 15.49% standard deviation, and the analysis of its confusion matrices revealed it to be an unreliable method for performing classification on our data. The combination of these two models has the potential to outperform both by overcoming their individual drawbacks.

The standard deviation decreased drastically, going as low as 1.36% with model-based evaluation and 3.61% with SVM-based. Its best mean accuracy surpassed both that of the CNN (80.72%) and the LSTM (80.44%), with 83.19%. The same is true for the mean accuracy.

It performed well according to its confusion matrices as well, outperforming the standard CNN in this regard and vastly improving on the shortcomings of LSTM. In conclusion, the combination of these two models resulted in a more consistent performance and increased accuracy. Table A3 shows the performance analysis of CNN + LSTM.

#### 3.3.4. ResNet

The Residual Neural Network produced the highest maximum mean accuracy out of all the methods: 84.23%. It also consistently kept to a low standard deviation, ranging from 1.36% to 2.83% for the model-based evaluation and from 4% to 7.72% for the SVM version. This model is the most complex, with the most number of layers, therefore it was expected that it would outperform smaller models such as the CNN or LSTM ones. The combined CNN + LSTM model performed comparatively well to ResNet, while the Convolutional Autoencoder fell short of these two. This suggests that our data scales in performance with model complexity.

The number of zero novice true positives for model evaluation was 11 out of 600, while for experts it is 110. In the case of SVM evaluation, these were four and seven, respectively. Regarding false positives, the number of cases with zero novice false positives were 115 for model-based evaluations and 19 for SVM-based, while for experts it was 170 for the model and 62 for SVM. It can be observed again that, even though in a strictly numerical sense the accuracy drops by using the SVM, it also makes the classification more precise. Table A4 shows the performance analysis of ResNet.

#### 3.3.5. convAuto

The only unsupervised learning method in the benchmark, the Convolutional Autoencoder, works on a completely different principle than the others. It predicts the classification labels using exclusively Support Vector Machines. It performs consistently and reliably, with a best mean accuracy of 79.77%. With 13 out of 600 cases of zero true positives for novices, 6 cases of zero for expert true positives, 26 cases of zero novice false positives and 17 cases of zero expert false positives, it provided one of the best results according to the confusion matrices, only outperformed by the Discrete Fourier and Discrete Cosine Transformations. Its efficacy could be improved by tuning its hyperparameters or employing an SVM kernel trick. Table A5 shows the performance analysis of convAuto.

#### 3.3.6. DFT

DFT and DCT are often evaluated side by side, and, even though their similarities seem to outweigh their differences at first sight, it is customary to investigate both, to see whether these seemingly small differences add up to significant differences in performance. The Discrete Fourier Transformation relies on a Support Vector Machine to classify the data based on the feature labels extracted with the help of the transformation. The results indicate that this method is not suited to evaluate OF-based data in this form because, even though its highest mean accuracy (73.83%) means that it has the potential to classify well about two thirds of the cases, overall it is closer to 50%, which means that it unable to make reliable predictions. DFT and DCT work with the assumption that, the more experienced someone is, the smoother their data’s time series is going to be represented in the frequency domain. This assumption holds well in the realm of kinematics, but OF may lack sufficient features or dimensions for this to hold true. Given our analysis, it would seem that these transformations depend on the quality rather than the quantity of data. Table A6 shows the overall performance of DFT.

#### 3.3.7. DCT

The Discrete Cosine Transformation works on similar principles to the DFT and also uses a Support Vector Machine for classification. The highest mean accuracy reached with this method was 78.61%, but similar to DFT it fell short in overall consistent accuracy. When comparing the two transformations, DCT has a clear edge over DFT—most likely due to its periodicity, which contrary to DFT does not introduce discontinuity. DCT outperformed DFT in all three metrics, and it gets close to being efficient enough, but it is still not suitable for reliable classification. Table A7 shows the performance analysis of DCT.

### 3.4. Results by Surgical Tasks

#### 3.4.1. Knot-Tying

Ming et al. found Knot-Tying to be the easiest surgical task to assess with both STIP and iDT [26]. Our method is similar in principle to their STIP method, as it also tracks the movement of interest points/features over time.

When it comes to the model evaluation, even the worst average accuracy (produced by LSTM) was at 74.75%. Regarding SVM evaluation, the same value was 62.1%. Coincidentall, y the highest SVM evaluated accuracy (80.43%) and the highest average SVM accuracy (76.07%) were also by LSTM. Given that model-evaluated LSTM results had many outliers, SVM could improve the recall and precision. For the best performing configuration of LSTM, 8 out of 25 trials (32%) had zero expert true positives. To measure the efficacy specifically for expert classification, the recall metric needs to be used.

(13) $$ExpertRecall=\frac{ExpertTruePositive}{NumOfExpertsInTrial}.$$

The highest individual expert recall of LSTM was 89.7%, but, given the eight cases where the value is zero, its mean is 24.36%.

**Figure 3:**
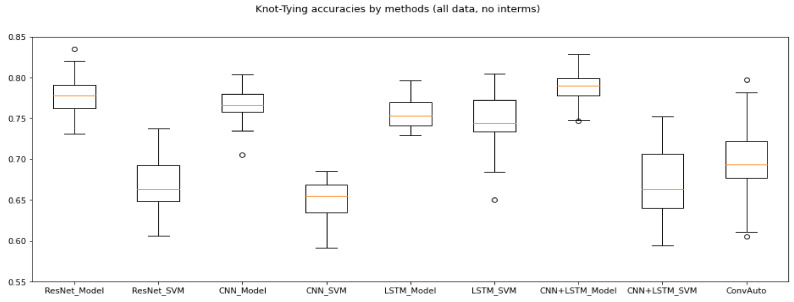
Knot-Tying accuracies without intermediates. These results (for all three skills) are computed using all possible sliding window parameters, while the comparison of two- and three-class classifications (presented in Table 2) compares the best performing data only. The best performing methods were ResNet and CNN + LSTM.

Overall, ResNet, CNN + LSTM and convAuto all performed well for the Knot-Tying task. LSTM has done well, but its low mean expert recall leaves the need for further investigation before it could be deemed as reliable, and CNN—considered to be a top-performer [21]—fell behind. Its highest accuracy was 80.42% and highest mean accuracy 78.38%. SVM predictions dropped its accuracy below 70%. ResNet’s model-based evaluation resulted in the highest accuracy (83.54%), while the highest mean accuracy (80.11%) came from the model-predictions of CNN + LSTM. Figure 3 shows the ranges of accuracies for each used method with Knot-Tying skill data.

#### 3.4.2. Suturing

**Figure 4:**
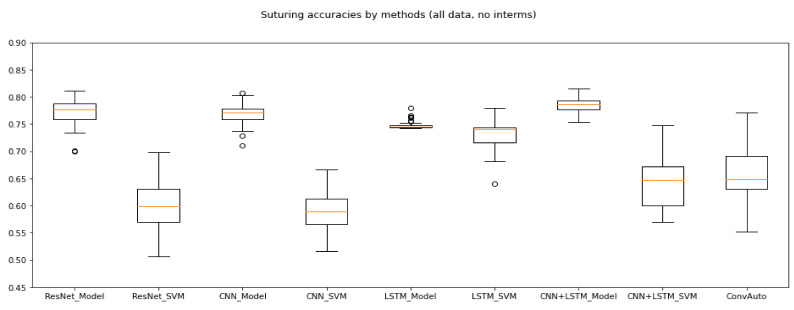
Suturing accuracies without intermediates. The best performing methods were ResNet, CNN and CNN + LSTM.

The same observations apply to Suturing as to Knot-Tying: ResNet, CNN + LSTM and convAuto performed well; LSTM showed high results in some cases, accompanied by confusion matrix anomalies (the maximum expert recall only being 3.33%, with an overall average of 0.22%); CNN seemingly performed the worst, even though it still did so above 75% on model average accuracies. Even though Ming et al. [26] also found that Suturing is harder to classify than Knot-Tying, CNN was found to be one of the most reliable methods by Yanik et al. in their review [21]. It is possible that the CNN model of the benchmark is too small, and it would perform better with higher complexity and more layers. Figure 4 illustrates the accuracy of each applied method, given the Suturing task.

#### 3.4.3. Needle-Passing

Ming et al. claimed that Needle-Passing was the hardest skill to perform classification for, since they did not find significant differences between the trajectories of expert and novice users’ left hand movements [26]. With our data generation method, only LSTM dropped significantly in efficacy in comparison with its performance on the other skills.

**Figure 5:**
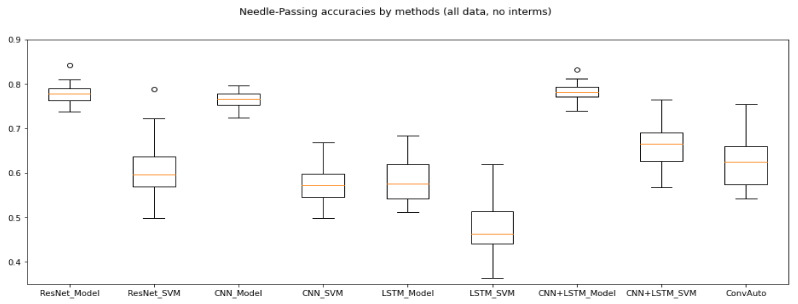
Needle-Passing accuracies without intermediates. ResNet, CNN and CNN + LSTM outperformed the other methods.

The highest Model average precision (79.74%) and the highest SVM accuracy (71.58%) were both achieved by CNN + LSTM, making it the overall best for the skill of Needle-Passing. The range of accuracies given all the Needle-Passing data for each method is illustrated in Figure 5.

### 3.5. Performance Analysis

Our generated data combined with the benchmark of Anh et al. [34] successfully outperformed the solutions of Ming et al. [26] in both Suturing and Knot-Tying, and they only slightly fell short in their results in Needle-Passing.

Our goal was to find a method that can achieve similar or better results to the state of the art in the field of surgical skill assessment, while keeping generalisation and practicality in mind, in order to keep it relevant for manual MIS training, where 3D and pose information is not available. It is important to note that the methods of Ahn et al. [34] were designed to be a benchmark, so it is not expected to outperform highly specialised, more complex models in the state of the art. The fact that our solution performed this accurately only goes to show the potential of OF-based visual features in skill assessment. The efficacy of classification can be improved with relative ease by building specialised networks for the task. Table 3 presents the detailed comparison of these methods.

Residual Neural Network performed the best, closely followed by the combined model of CNN and LSTM. DFT and DCT performed too poorly to be able to rely on them for classification. Even though LSTM’s accuracy is high, its confusion matrices showed it to be unreliable.

Based on the results, we can conclude that the Residual Neural Network performs the best in our video data-based surgical skill assessment approach, closely followed by the combined model of CNN and LSTM. The Discrete Fourier and Discrete Cosine Transformations performed too poorly to be able to rely on them for classification. Contrary to its relatively high accuracy ratings, the simple LSTM model was found to be unreliable with the generated data employed.

The first and fourth trial of each run—meaning the one where the first or fourth attempt of each user was employed for the test set—produced the best results. Regarding model-based evaluation, the first trials performed consistently above 80%: 70% of these trials had accuracy over 85%, and 36.67% of these had at least 90%, with 95.74% being the highest. SVM-based evaluations—including convAuto, DFT and DCT—had 10 results above 90% in these trials, one of which was by convAuto, and they provided 26 results above 85%, with 94.68% being the highest accuracy.

Similarly, in the fourth trials of each run, there were 21 results above 85%, 18 of which were above 90% with the model-based predictions, and 27 results above 85%, 23 of which were above 90% with the Support Vector Machines. The maximum achieved accuracy was 96.13% for model predictions and 95.7% for SVM evaluation. The second, third and fifth trials also produced accuracies above 90% occasionally, but much less often.

It was observed that, although the SVM classification decreases the overall accuracy, it performs more consistently well overall. This is likely due to the fact that SVMs are susceptible to outliers [43]. They perform well in high-dimensional feature spaces, and, although we have 240 features, the number of rows greatly outnumbers this.

## 4. Discussion

### 4.1. Kinematics vs. Visuals

Kinematic data are highly precise and describe the motions of each relevant joint with the combination of rotation matrices, linear and angular velocities. Their complexity and feature-richness is the general complexity of most visual features. However, visual solutions have the potential to replace kinematic ones due to the fact that, theoretically, it is possible to calculate the approximation of the same kinematic data from visual input [44].

Images are inherently 2D in nature, therefore visual methods are restricted to two dimensions as well. This can be counteracted by 3D visual methods, but they require camera calibration information and stereo recording, which are not available during traditional MIS trainings, where skill assessment is of the highest importance. However, given their accessibility in RAMIS skill assessment, and their higher performance, their application is justified. In 2019, Funke et al. [25] achieved close to 100% accuracy on all three surgical skills in the JIGSAWS set, using 3D visual features, something previously only possible with kinematic solutions. There seems to be, however, a research gap in the use of 2D-only visual features, which is why this work’s focus was chosen as such.

The main focus of this work was to evaluate the visual input in terms of its efficacy, i.e., the accuracy such data can produce in comparison to the results of Anh et al. [34]—using the exact same benchmark methods they used with kinematic data, so the performance of our OF-based solution can measure up to their kinematic benchmark directly. It presents a general, easily scalable and practical way of acquiring numerical data out of any surgical video recording. It is generally applicable, as any video recording can be used with the proposed ROI-selecting technique, and it is scalable, as the code can be easily tailored to specific tasks to accommodate any number of surgical tools—even customisable runtime. For the purpose of this work, the number of tools was explicitly set to two, as the data of JIGSAWS only operated on two tools. Its efficacy is currently way below that of the kinematic solutions, and it possibly will not ever be as accurate as kinematics can be, but with additional features the accuracy can be improved, and its benefits of general applicability outweigh any drawback it might have against the kinematic systems. Moreover, visual solutions have the potential to replace kinematic ones due to the fact that, theoretically, it is possible to calculate the approximation of the same kinematic data from visual input [44].

### 4.2. Segmentation, Surgical Tool Tracking

A significant bottleneck of using visual features as data in surgical skill assessment is the necessity of an accurate universal segmentation method. Surgical tools vary in shapes and sizes, and, for more precise assessments, where rather than just analysing the movement of the tools, it is also relevant which tool performs which subtask with what level of precision, the semantic segmentation of surgical tools comes in as a basic requirement. For this work, segmentation was not necessary for our goal, which was to create a proof of concept for 2D visual features in surgical skill assessment. In the long term, however, in order to be able to generate tool trajectories for skill assessment, the use of a reliable, generally applicable segmentation method is unavoidable, which is why some of the state-of-the-art segmentation solutions are investigated and introduced in the Appendix A, providing context on this crucial related field. Here, however, we mainly discuss the limitations and utility of the ROI-selector method described above.

#### Limitations of the ROI Selector

The ROI-selector method presents a trade-off between full automation and generalisation. Segmentation and tracking of surgical tools is a precarious technique and hard to generalise, and therefore the deep learning methods need to be specifically trained for each use-case, or at the very least for the specific instruments used for the task.

With the ROI-selector method, the video can be quickly and conveniently converted to the necessary format. The current version is built for the tracking of two tools—as it was needed for the JIGSAWS videos—but it could be easily repurposed to be able to handle any number of objects. This enables the use of this method also in traditional MIS training, which is the most important aspect of surgical skill assessment, given that for RAMIS one needs to be an experienced surgeon to begin with. In its current state, however, there is no guarantee that the tracking does not lose the object if it leaves the frame, and neither does it follow new instruments that appear at later stages in the video. Solutions similar to the previously introduced automatised laparoscope control of Li et al. [45] could ensure that the tools do not leave the field-of-view, but the implementation of a resampling method would be a more reliable solution.

Further challenges in tracking include the diversity of background environment, glistening surfaces and movement in the background itself, as well as the movement of the camera, the metal tools reflecting light, bleeding, bloodstains on tools, occlusions and smoke, coming from cauterising injuries [46,47,48].

## 5. Conclusions

This work aimed to create a generally applicable solution that can enable the creation of visual-based surgical skill assessment methods and can potentially lead to the inclusion of automated skill assessment in the curriculum of minimally invasive surgical training, introducing benefits such as objectivity, reproducibility and the fact that it would not require human expertise.

The proposed method measured up to the state-of-the-art approaches in 2D visual-based skill assessment, with over 80% accuracy for all three surgical subtasks available in JIGSAWS (Knot-Tying, Suturing and Needle-Passing). By introducing new visual features (such as image-based orientation and image-based collision detection), employing other SVM kernel methods, tuning the hyperparameters or using other methods (such as the boosted tree algorithm), classification accuracy can be further improved.

We showed the potential use of optical flow as an input for RAMIS skill assessment, highlighting the maximum accuracy achievable with these data by evaluating it with an established skill assessment benchmark, by evaluating its methods independently. The highest performing method, the Residual Neural Network, reached 81.89%, 84.23% and 83.54% accuracy for the skills of Suturing, Needle-Passing and Knot-Tying, respectively.

## Figures and Tables

**Table 1:** The misclassification rate of Intermediate users with each method, as shown by the Intermediate True Positive (TP) values.

Method	# of Zero TP Cases	Percentage of 0 TPs
ResNet	42 (out of 150)	28%
CNN	47 (out of 150)	31.33%
CNN + LSTM	55 (out of 150)	36.66%
LSTM	117 (out of 150)	78%
convAuto	12 (out of 75)	16%

**Table 2:** The performance overview of each method in the case of two and three classes, respectively.

Method	Eval.	Class Num.	Min. Run	Max. Run	Min. Trial	Max. Trial
CNN	Model	2	73.7%	79.12%	54.34%	93.67%
		3	49.6%	66.83%	35.69%	75.44%
CNN	SVM	2	53.97%	66.53%	29.45%	94.68%
		3	28.15%	56.8%	43.65%	70.59%
LSTM	Model	2	51.16%	79.62%	24.44%	90.64%
		3	40.48%	62.19%	15.25%	69.63%
LSTM	SVM	2	40.95%	80.44%	22.22%	91.94%
		3	30.2%	60.87%	15.25%	70.86%
CNN+ LSTM	Model	2	74.69%	83.19%	56.42%	93.65%
		3	56.12%	73.09%	46.37%	82.65%
CNN+ LSTM	SVM	2	58.987%	73.44%	28.27%	93.23%
		3	33.12%	68.57%	5.15%	82.94%
ResNet	Model	2	73.75%	83.54%	54.55%	95.74%
		3	47.93%	70.25%	33.46%	80%
ResNet	SVM	2	54.21%	73.64%	23.3%	93.04%
		3	25.92%	61.5%	7.15%	79.17%
convAuto	SVM	2	58.58%	75.52%	33.33%	93.21%
		3	30.82%	52.57%	16.14%	77.36%

**Table 3:** The comparison of our results to that of the state of the art. Every method listed here used the JIGSAWS dataset as visual input data source.

Author (Year)	Method	ST	NP	KT
Funke et al. (2019)	3D ConvNet +TSN [25]	100%	96.4%	95.8%
Ming et al. (2021)	STIP [26]	79.29%	87.01%	72.57%
Ming et al. (2021)	iDT [26]	76.79%	83.81%	76.65%
Our solution	CNN [34]	80.72%	79.66%	80.41%
Our solution	CNN + LSTM [34]	81.58%	83.19%	82.82%
Our solution	ResNet [34]	81.89%	84.23%	83.54%

**Figure A1:**
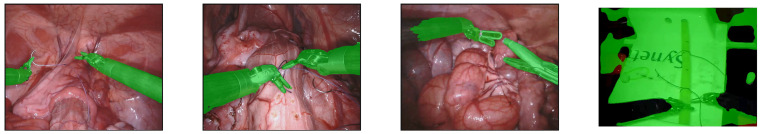
Example outputs of the MICCAI challenge winning surgical tool segmentation solution on its own data, and the output on a frame from JIGSAWS (right). This example highlights the issue of overfitting and the lack of generalisation capability of deep learning techniques in surgical instrument segmentation.

## Data Availability

Publicly available datasets were analyzed in this study. This data can be found here: https://github.com/SimonNgj/compssa, accessed date: 30 June 2021. https://github.com/ternaus/robot-surgery-segmentation, accessed date: 30 June 2021. https://github.com/ABC-iRobotics/VisDataSurgicalSkill, accessed date: 30 June 2021.

## Appendix A. State-of-the-Art Segmentation Methods

### Appendix A.1. YOLO

YOLO (You Only Look Once) was published by Redmon et al. in 2016 [49], and, since then, it has become a standard of object detection research. Building on the original algorithm, Redmon published an improved version, YOLOv3, in 2018 [50].

Traditional object detection methods use specialised localisers or classifiers, processing the input at different scales and positions, then scoring regions of the image, ultimately outputting the highest scoring areas as detections. YOLO, however, as the name suggests, aims to increase detection speed by processing the input only once. A single network is applied to the entire picture, dividing it into regions, predicting bounding boxes and the corresponding probability of each region. This approach deals with the input in a global context and is therefore not affected by any local bias.

Its most remarkable quality is that it uses a single neural network, contrary to the thousands of networks required by the previously state-of-the-art Region-Based Convolutional Neural Networks (R-CNN) [51]. This makes YOLO more than one thousand times faster than R-CNN, and even one hundred times faster than its improved version, Fast R-CNN [52]. Speed is not the only quality of YOLO, however, as it achieves real-time inference while keeping up with the accuracy of other methods. YOLOv3 also offers easily customisable speed and accuracy tradeoff by changing to a different sized model without the need to retrain.

As a reliable, accurate and extremely quick, real-time object detection method, YOLO can most likely be of use in further research even in specialised fields such as surgical skill assessment; it just requires the specialised training and testing data, the creation and labelling of which is a long and tedious undertaking. The publication of Li et al. [45] is a good example of utilising the potential of YOLOv3 for surgical tool tracking. Their system approaches the segmentation of surgical tools from a very specific use-case by introducing an autonomous surgical instrument tracking for robot-assisted laparoscopic surgery, using so-called visual tracking space vectors in conjunction with a modified YOLOv3. First, they use the space vectors to track and follow the instruments with the laparoscopic camera, so that the precision of visual feedback can be optimised. The modified YOLOv3 is simultaneously used to detect, segment and classify the tracked tools. The proposed method automatically adapts to changes in the number of visible surgical instruments, does not require depth information and accurately and reliably controls the laparoscope by controlling the field-of-view. They claimed that, despite their highly accurate performance, the computational complexity of previous state-of-the-art deep learning-based methods such as Fast R-CNN present unfavourable conditions for automated laparoscope control. That is why they ultimately settled on using YOLOv3 to detect the surgical tool’s tips. The modified YOLOv3 does not require additional markers and is allegedly unaffected by occlusions and blood contamination.

### Appendix A.2. Ternausnet

In 2017, the Medical Image Computing and Computer Assisted Intervention (MICCAI) society [53], as part of their Endoscopic Vision Challenge [54], called for a Robotic Instrument Segmentation Sub-Challenge [55], the winning solution of which [47] was made publicly available as a repository at https://github.com/ternaus/robot-surgery-segmentation (access date: 30 June 2021).

Azqueta-Gavaldon et al. investigated the use of augmented training data in surgical tool segmentation [56]. Although they found that the method works well for training neural networks, and it saves a lot of time by automating the labelling process usually done by hand, they also noted that the model they used could not generalise well to surgical tools with different shapes than the ones it was trained for. As shown in Figure A1, this applies to the solution of Shvets et al. [47] as well. Even though their results are exceptional, securing them first place in multiple sub-categories of MICCAI’s vision challenge, running their segmentation method on frames from JIGSAWS falls short of expectations.

The dataset provided for the EndoVision challenge consists only of eight videos, each 225 frame long. They were recorded using a da Vinci Xi system, during a set of different procedures, performed on swine carcasses. Seven distinct surgical tools were used during the recordings, and Shvets et al. managed to train their networks to identify all seven of them individually. Due to the difference in background scenery and tool shapes, as well as the fact that the training data were very restricted, their network failed to segment the surgical tools in the examined JIGSAWS videos.

Our findings show that, no matter how well a network is proven to perform on a given dataset, if the training data are not suitable to the specific task we need it to segment, pre-trained solutions are unable to generalise well enough and will not work.

## Appendix B. Performance Analysis Tables

**Table A1:** Overall performance of CNN, given each parameter configuration. The 150 × 30 configuration had the best run, the best mean performance was by 120 × 30, the lowest standard deviation came from 120 × 60 and the best trial was from 150 × 60 (all in bold). It generally seems to scale with more data (higher window and lower step sizes), but it is not exactly linear.

Parameters	Evaluation	Mean	Standard Deviation	Best Run	Best Trial
180 × 30	Model	77.05%	2.52%	80.42%	92.26%
180 × 30	SVM	58.91%	5.02%	67.99%	92.26%
180 × 60	Model	75.79%	2.56%	79.76%	92.36%
180 × 60	SVM	58.52%	4.24%	64.77%	90.45%
150 × 30	Model	77.04%	2.09%	**80.72%**	93.33%
150 × 30	SVM	60.08%	5.28 %	68.5%	93.33%
150 × 60	Model	76.47%	1.49 %	79.12%	93.67%
150 × 60	SVM	61.36%	3.28%	66.53%	**94.68%**
120 × 30	Model	**77.19%**	1.7%	80.25%	93.1%
120 × 30	SVM	61.65%	4.3%	68.31%	92.8%
120 × 60	Model	76.34%	**1.43%**	78.16%	94.44%
120 × 60	SVM	60.33%	5.49%	68.17%	91.98%
90 × 30	Model	77.15%	1.79%	79.82%	92.62%
90 × 30	SVM	61.91%	4.72%	68.27%	92.31%
90 × 60	Model	76.41%	1.64%	78.79%	93.25%
90 × 60	SVM	59.38%	4.57%	66.48%	92.02%

**Table A2:** Overall performance of the Long Short-Term Memory model, given each parameter configuration. This method learns patterns over time, so it requires the data to be a time series. Its performance does not seem scale with more data, as the difference between different parameters seems to be marginal. Best accuracies are highlighted in bold.

Parameters	Evaluation	Mean	Standard Deviation	Best Run	Best Trial
180 × 30	Model	67.98%	10.76%	79.62%	90.65%
180 × 30	SVM	64.54%	15.35%	**80.44%**	91.94%
180 × 60	Model	**71.56%**	**5.97%**	79.44%	95.36%
180 × 60	SVM	65.53%	11.98%	78.34%	87.89%
150 × 30	Model	69.09%	9.13%	77.899%	92.73%
150 × 30	SVM	67.06%	11.12%	77.899%	92.73%
150 × 60	Model	70.23%	7.45%	77.07%	83.33%
150 × 60	SVM	66.45%	12.47%	77.74%	86.03%
120 × 30	Model	68.65%	10.07%	76.93%	92.81%
120 × 30	SVM	63.895%	15.49%	78.3%	94.06%
120 × 60	Model	70.35%	7.98%	79.19%	89.86%
120 × 60	SVM	64.66%	13.21%	77.82%	87.77%
90 × 30	Model	69.04%	8.73%	76.19%	83.15%
90 × 30	SVM	63.64%	13.64%	76.43%	86.46%
90 × 60	Model	70.698%	8.3%	78.66%	**95.71%**
90 × 60	SVM	63.75%	14.296%	78.49%	**95.71%**

**Table A3:** Overall performance of CNN + LSTM, given each parameter configuration. The standard deviation not going above 5% in any case, combined with the generally high accuracy values, means that this method performed well overall, given both evaluation types and across all the different data configurations. Best accuracies are highlighted in bold.

Parameters	Evaluation	Mean	Standard Deviation	Best Run	Best Trial
180 × 30	Model	79.14%	1.85%	**83.19%**	93.65%
180 × 30	SVM	65.91%	4.1%	73.44%	93.23%
180 × 60	Model	**79.19%**	1.697%	82.82%	93.3%
180 × 60	SVM	67.77%	4.42%	75.84%	**95.88%**
150 × 30	Model	78.59%	**1.36%**	81.58%	92.38%
150 × 30	SVM	65.82%	5.1%	73.12%	92.06%
150 × 60	Model	78.33%	1.69%	81.22%	92.5%
150 × 60	SVM	65.8%	4.68%	73.39%	89.87%
120 × 30	Model	78.14%	1.69%	81.02%	90.97%
120 × 30	SVM	64.27%	4.18%	75.23%	92.03%
120 × 60	Model	78.899%	1.48%	81.55%	94.29%
120 × 60	SVM	67.47%	4.12%	74.74%	90.74%
90 × 30	Model	77.14%	1.74%	80.5%	92.31%
90 × 30	SVM	61.99%	3.61%	67.89%	92%
90 × 60	Model	78.37%	1.51%	80.77%	94%
90 × 60	SVM	66.69%	4.92%	76.48%	92.02%

**Table A4:** Overall performance of the Residual Neural Network model, given each parameter configuration. One of the best methods according to our data, it performed consistently and accurately all over the board. Best accuracies are highlighted in bold.

Parameters	Evaluation	Mean	Standard Deviation	Best Run	Best Trial
180 × 30	Model	77.79%	2.08%	80.75%	96.13%
180 × 30	SVM	62.43%	4%	71.16%	91.94%
180 × 60	Model	78.07%	2.43%	**84.23%**	**96.39%**
180 × 60	SVM	62.83%	5.88%	73.03%	91.72%
150 × 30	Model	76.67%	2.56%	81.08%	94.98%
150 × 30	SVM	62.46%	5.42%	72.21%	91.43%
150 × 60	Model	**78.65%**	2.16%	83.54%	95.74%
150 × 60	SVM	62.84%	5.35%	73.64%	93.04%
120 × 30	Model	76.56%	1.72%	79.95%	92.5%
120 × 30	SVM	62.01%	5.2%	72.82%	92.81%
120 × 60	Model	77.66%	2.22%	81.996%	91.36%
120 × 60	SVM	61.98%	5.58%	70.69%	90.12%
90 × 30	Model	76.21%	2.83%	80.77%	92.62%
90 × 30	SVM	63.28%	7.72%	78.85%	92.31%
90 × 60	Model	78.62%	**1.36%**	81.41%	93%
90 × 60	SVM	62.19%	6.33%	72.84%	92.02%

**Table A5:** Overall performance of the Convolutional Autoencoder, given each parameter configuration. Its mean accuracy is consistently above 70%, the standard deviation is below 6.5% and, although it was outperformed by the supervised methods, it showed itself to be a reliable method. Best accuracies are highlighted in bold.

Parameters	Mean	Standard Deviation	Best Run	Best Trial
180 × 30	67.16%	5.03%	74.79%	90.96%
180 × 60	64.01%	5.16%	71.95%	87.22%
150 × 30	65.84%	6.8%	**79.77%**	91.03%
150 × 60	66.08%	**4.67%**	71.38%	90.3%
120 × 30	64.64%	5.54%	78.14%	**95.48%**
120 × 60	**67.78%**	4.75%	75.52%	93.21%
90 × 30	64.64%	6.31%	74.24%	90.95%
90 × 60	66.41%	6.15%	77.097%	93.25%

**Table A6:** Overall performance of the Discrete Fourier Transformation, given each parameter configuration. Outperformed by DCT in every regard, this was one of the least suitable methods for the data generated. Best accuracies are highlighted in bold.

Parameters	Mean	Standard Deviation	Best Run	Best Trial
180 × 30	58.88%	13.3%	**73.83%**	**78.47%**
180 × 60	55.22%	11.6%	67.67%	72.02%
150 × 30	**59.38%**	8.46%	69.01%	78.14%
150 × 60	58.41%	**4.25%**	62.88%	73.08%
120 × 30	56.02%	7.38%	64.1%	71.88%
120 × 60	53.64%	4.77%	58.38%	72.81%
90 × 30	48.35%	5.23%	54.28%	59.74%
90 × 60	52.16%	5.45%	58.4%	68.13%

**Table A7:** Overall performance of the Discrete Cosine Transformation, given each parameter configuration. Although the highest mean accuracy was the result of 180 × 30, i.e., the biggest files, the most consistent performance—meaning the lowest standard deviation—was achieved using the smallest files (generated with a 90 × 30 sliding window). This indicates that DCT’s performance scales with data, but, without suitable features, its accuracy deviates more. Best accuracies are highlighted in bold.

Parameters	Mean	Standard Deviation	Best Run	Best Trial
180 × 30	**61.81%**	13.71%	**78.61%**	**84.67%**
180 × 60	59.91%	12.44%	75.12%	81.16%
150 × 30	60.83%	11.12%	75.13%	81%
150 × 60	58.09%	10.1%	70.71%	78.68%
120 × 30	57.12%	7.67%	67%	75.35%
120 × 60	55.74%	7.36%	65.11%	76.53%
90 × 30	52.89%	6.36%	60.79%	72.76%
90 × 60	57.66%	**3.09%**	60.78%	68.13%
